# Addressing the Needs of the Whole Child: What Public Health Can Do to Answer the Education Sector’s Call for a Stronger Partnership

**Published:** 2011-02-15

**Authors:** Diane Allensworth, Theresa C. Lewallen, Beth Stevenson, Susan Katz

**Affiliations:** Centers for Disease Control and Prevention; ASCD, Alexandria, Virginia; Centers for Disease Control and Prevention, Atlanta, Georgia; Centers for Disease Control and Prevention, Atlanta, Georgia

## Abstract

Although the overall level of child health in the United States remains high, public health professionals know that racial and ethnic disparities in child and adolescent health persist and that lifestyle choices related to chronic disease in adults are often established in childhood and adolescence. And yet, those health needs are not the public health sector's alone to resolve. We have natural partners among educators. Improving graduation rates is one of the most cost-effective ways to reduce health disparities. This article provides strategies for how public health professionals can answer this call by educators to address the needs of the whole child.

## Background

Education and health are interdependent systems that increasingly need to collaborate in helping our nation's children. The authors of *The Learning Compact Redefined: A Call to Action* recommend that local schools work closely with the public health community to adequately address the conditions that affect learning (1). This call represents both a challenge and an opportunity for the public health community. Not only do public health data indicate that education levels and health outcomes are highly correlated but public health professionals also have pressing needs to reach students to achieve health outcomes.

Health and education are integrally linked (2). Children who do not complete high school are likely to become adults who have employment problems, lower health literacy, higher rates of illness, and earlier deaths than those who graduate from high school (3,4). Evidence suggests that improving high school graduation rates may be more cost-effective than most medical interventions in reducing health disparities (3,5). Graduation from high school is associated with an increase in average lifespan of 6 to 9 years (6). The reasons students drop out of school are complex (7), and health can be integrally related to many of these reasons, including barriers to learning such as hunger and poor nutrition and even fear for safety at school (8). Health problems contribute to absenteeism and, in turn, absenteeism (9) as well as unintended pregnancy and delinquency (5) are associated with dropping out of school. Other risk factors for dropping out are frequent changes of schools, lack of parent participation in schooling, and nonproductive use of leisure time, such as watching many hours of television daily (8).

The United States ranks 18th among nations in high school completion rates (10). Every school day, 7,000 students drop out of school, resulting in 1.2 million dropouts annually (3). Although generally the percentage of students in the United States who complete school is close to 70%, the rates for poor Native American, African American, and Hispanic students are substantially lower. In some urban areas the number of Hispanic and African American male students who graduate is less than 50% (9).

Dropping out of school contributes to future unemployment or underemployment, and dropouts are more likely to commit crime or rely on government assistance for health care, housing, and food. Dropouts are less likely to raise healthy, well-educated children (3). A combination of underlying health, family, community, and education issues must be addressed to prevent this cycle. No one sector can address the complexity of the interdependent needs of children. Previous studies have found that when the public health and education sectors work together and collaborate with community agencies, students' academic achievement and health improve (11-14).

## Educators Request Public Health Involvement

Educators recognize that a focus on academic achievement as a means to ensure graduation requires a concomitant focus on the areas that support learning — including safety and physical, mental, and social health. Research indicates that students are more successful in school and in life when they experience a broad, challenging, and engaging curriculum; when they feel connected to their school and surrounding community; when their physical and emotional health is supported; and when schools offer safe and nurturing environments (15-22). By providing these conditions, schools and the community address the cognitive, physical, social, and emotional well-being of the whole child and support children's growth and development into knowledgeable, healthy, and productive adults.

To address these conditions for supporting children, in 2006, ASCD, a professional educational association (formerly The Association for Supervision and Curriculum Development), commissioned an interdisciplinary panel that included public health leaders to develop *The Learning Compact Redefined: A Call to Action* (1). This report challenges schools and communities to work together in new ways to develop "successful learners who are knowledgeable, emotionally and physically healthy, civically active, engaged, prepared for economic self-sufficiency, and ready for the world beyond formal schooling (23)." *The Learning Compact Redefined: A Call to Action* (1) is designed around the following 5 elements for the nation's students:

1. Each student enters school healthy and learns about and practices a healthy lifestyle.
2. Each student learns in an intellectually challenging environment that is physically and emotionally safe for students and adults.
3. Each student is actively engaged in learning and is connected to the school and broader community.
4. Each student has access to personalized learning and to qualified and caring adults.
5. Each graduate is prepared for success in college or further study and for employment in a global environment.

These 5 elements provide a framework for how health and education can begin to work together to achieve mutual goals.

## A Public Health Rationale for an Expanded Partnership

Schools are one of the most efficient systems for reaching children and youth to provide health services and programs, and approximately 95% of all US children and youth attend school (24). Establishing healthy behaviors during childhood is easier and more effective than trying to change unhealthy behaviors during adulthood and affords the population more years of healthy life. Schools play a critical role because of these factors:

- Each school day is an opportunity for the nation's 55 million students to learn about health and practice the skills that promote healthy behaviors.
- The nation's 125,000 schools have the potential to provide opportunities for students to practice healthy behaviors such as eating healthy foods and participating in physical activity (25).

To illustrate the relationship between health and education from the perspective of *The Learning Compact Redefined: A Call to Action,* we developed a model that portrays some of the factors that can affect the health and education of children and youth (Figure 1). *The Learning Compact Redefined: A Call to Action* recognizes that health and education are interdependent and can result in better health and education outcomes, increased graduation rates, and ultimately, healthier adults who will have healthier children. The model describes an interdependent process where increases in which high school graduation rates are linked to improved health outcomes.

**Figure:**
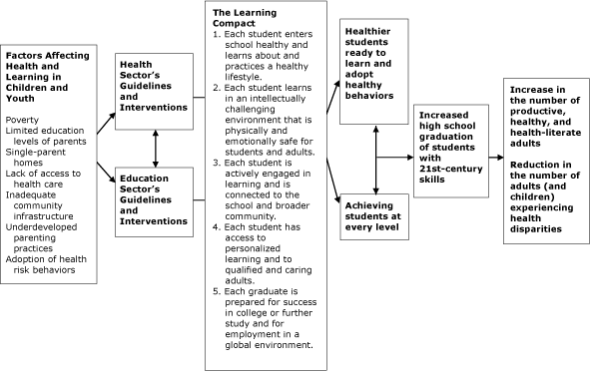
The Learning Compact for Children can link the health sector and the education sector in a collaborative effort that addresses the social determinants of health and promotes better learning and health outcomes for children and the adults that they will become.

**Table:** 

The logic model is a diagram of text boxes with arrows between them, leading the reader from left to right.
The logic model begins with a box entitled “Factors Affecting Health and Learning in Children and Youth.” Those factors are poverty, limited education levels of parents, single-parent homes, lack of access to health care, inadequate community infrastructure, underdeveloped parenting practices, and adoption of health risk behaviors. These factors lead to the development of guidelines and interventions by the health sector and the education sector. These guidelines and interventions lead to the development of “The Learning Compact,” which includes the following 5 components:
1. Each student enters school healthy and learns about and practices a healthy lifestyle.
2. Each student learns in an intellectually challenging environment that is physically and emotionally safe for students and adults.
3. Each student is actively engaged in learning and is connected to the school and the broader community.
4. Each student has access to personalized learning and to qualified and caring adults.
5. Each graduate is prepared for success in college or further study and for employment in a global environment.
The Learning Compact, as proposed, should lead to outcomes in the health and education of the nation’s students. The outcomes of the compact are 1) healthier students ready to learn and adopt healthy behaviors; 2) achieving students at every level; 3) increased high school graduation of students with 21st-century skills; 4) an increase in the number of productive, healthy, and literate adults; and 5) a reduction in the number of adults (and children) experiencing health disparities.

## Strengthening the Partnerships Between Health and Education

Although school districts and health departments have historically worked together and best practices from coordinated school health program examples encourage schools to involve community agencies (26), it is not standard in many communities for the public health department to provide services to schools. A 2008 survey of local health department jurisdictions found that only 36% of these jurisdictions conducted school health activities (27).

Despite challenges to collaborative work between public health and education, both sectors are adept at partnerships, and successes are emerging with demonstrated partnership activities. The interdisciplinary panel that developed *The Learning Compact* has recommended that public health and education sectors in every community discuss steps to ensure that the conditions for learning are met. For the public health sector, this conversation must begin with the understanding that today's educational practice and policies focus overwhelmingly on academic achievement. The National Association of State Boards of Education's resource *How Schools Work and How to Work with Schools* provides questions that public health professionals can ask to begin the conversation using education-focused questions (28).

- What do we want from schools?
- What can we offer schools?
- Whom should I contact within the education system?
- How is our agenda related to what's important to education leaders?

Similarly, educators should ask what they need from public health, what education can offer, and how health and education agendas can work together to reach mutual health and education outcomes.

We present strategies that public health agencies could use to support education and to address the 5 elements of *The Learning Compact* (Table 1). Strategies to be implemented would be based on conversations between the 2 sectors.

Since *The Learning Compact* was published, ASCD has supported partnership initiatives between education and public health that are based on an understanding of the interdependent nature of education and health outcomes and driven by the 5 elements of the learning compact. ASCD conducted a 3-year evaluation of its 11 Healthy School Communities pilot sites, which looked at how well those sites addressed the principles of *The Learning Compact.* Student outcomes improved at sites where the school and public health agencies worked together to meet their mutual goals. For example, in Des Moines, New Mexico, and in Indianapolis, Indiana, school-based health care and wellness centers provide services to students, their families, and community members. Student attendance and discipline referrals — factors that correlate with graduation rates — have improved since the establishment the health centers. In Hills, Iowa, where access to fresh produce is limited, the public health department provides health education as well as fruits and vegetables in a low-income elementary school. At the same time, the school implemented a research-based approach to improving school climate. The school reports fewer discipline problems and improvement in reading and math, which is attributed to students' better nutrition and changes to the school climate. Other public health relationships of the Healthy School Communities pilot sites include the school district and public health agency sharing the cost of providing nurses in the schools for health screenings and referrals and coordinating the district's implementation of an evidence-based health program (29). The key factor in each of these partnerships is the role that the public health staff played in the school improvement process — working with the school improvement team to make systemic, sustainable change in the school environment through policy and practice that align with the schools' mission, vision, and goals (RF Valois, unpublished report, 2009).

## Summary

*The Learning Compact Redefined: A Call to Action* recommends that local schools work closely with the public health community to address conditions that affect learning. Not only do public health data indicate that education levels and health outcomes are highly correlated but health officials also have pressing needs to address the current health needs of children as well as to promote behaviors that will affect health throughout their lifespan. Education and health are interdependent systems that urgently need to collaborate in helping our nation's children. Together, public health and education can reduce absenteeism, improve achievement, and increase graduation rates. In turn, improved graduation rates will help reduce health disparities and increase the quality and years of healthy life — 2 major goals of the public health sector and the nation. Such outcomes can help curtail the intergenerational cycle of poverty that is the underlying factor for many public health problems. The public health sector has a vital role to play in responding to the call from educators.  We can no longer afford to consider the work of public health and education as separate paths to our respective outcomes. The 5 elements of the learning compact provide a framework for education and health to work more closely together to address the health and education needs of all students.

## Figures and Tables

**Table.** Roles for Public Health Agencies in Supporting Local Education Agencies

**Conditions for Learning from *The Learning Compact Redefined: A Call to Action***	**Role for Public Health Agencies**
1. Each student enters school healthy and learns about and practices a healthy lifestyle.	Ensure public health involvement on district-level school health councils and public health support for school-level health teams. Ensure that all children who qualify for the State Children's Health Insurance Program are enrolled. Ensure all students and their families have a medical home with access to health services. Promote a quality school health program. Provide visits by nurse to teen mothers to establish healthy starts for vulnerable children. Encourage schools to implement the CDC guidelines (eg, tobacco use prevention, physical activity and nutrition, safety and violence prevention, food safety). Promote the use of a sequential, comprehensive health education curriculum that meets the *National Health Education Standards.* Promote the use of a sequential physical education curriculum that meets recommended standards: *Moving into the Future: National Standards for Physical Education,* 2nd edition. Promote the use of school health assessments (eg, CDC's *School Health Index*, ASCD's *Healthy School Report Card*) to initiate a program planning process for continuous improvement at each school. Participate on school improvement planning teams. Promote community surveillance to identify health-related absenteeism, health risk behaviors of students and adults, and health protective factors of students. Promote programs that aggressively reduce preventable absenteeism — kindergarten through high school.
2. Each student learns in an intellectually challenging environment that is physically and emotionally safe for students and adults.	Assure safe, nonviolent schools by promoting the adoption of CDC's *School Health Guidelines to Prevent Unintentional Injuries and Violence*. Promote establishment of the healthy physical environment by using EPA's *Healthy SEAT* assessment. Ensure that the curriculum has a scope and sequence in which all students learn personal and social skills. Promote engaging business, health care, community service, and faith-based sectors on district school health councils and local school health teams. Support creation and implementation of school and community youth development programs. Include schools in the community public health planning process. Promote worksite wellness initiatives for school staff. Encourage schools to open the worksite health promotion programs to students and families in the community.
3. Each student is actively engaged in learning and is connected to the school and broader community.	Encourage the agencies on the district school health council to provide learning opportunities for students in community agencies. Collaborate with out-of-school programs to ensure access to quality prevention programs and health services. Engage students as partners on public health planning teams and in designing public health interventions for students, their families and the community at large. Engage students and community in mentoring and life-enriching opportunities (eg, service learning, peer education, cross-age mentoring, youth development).
4. Each student has access to personalized learning and to qualified and caring adults.	Work with schools and communities to develop service learning and out-of-school learning opportunities. Advocate for the development of effective mentoring programs encouraging agencies on the school health council to provide mentors and tutors, matched to student vulnerabilities.
5. Each graduate is prepared for success in college or further study and for employment in a global environment.	Promote the use of the National Health Education Standards and the National Physical Education Standards for the development of school and out-of-school health and physical education programs. Provide information to schools on the role health literacy plays in preparation for success in life.

Abbreviations: CDC, Centers for Disease Control and Prevention; EPA, Environmental Protection Agency.
